# Quantitative behavioural phenotyping to investigate anaesthesia induced neurobehavioural impairment

**DOI:** 10.1038/s41598-021-98405-x

**Published:** 2021-09-29

**Authors:** Pratheeban Nambyiah, Andre E. X. Brown

**Affiliations:** 1 Institute of Clinical Sciences, Faculty of Medicine, Imperial College London, London, UK; 2 MRC London Institute of Medical Sciences, London, UK; 3 Present Address: Department of Anaesthesia, Great Ormond Street Hospital for Children, London, UK

**Keywords:** Developmental neurogenesis, Disease model, Cell death in the nervous system, Neurodegeneration, Paediatric research

## Abstract

Anaesthesia exposure to the developing nervous system causes neuroapoptosis and behavioural impairment in vertebrate models. Mechanistic understanding is limited, and target-based approaches are challenging. High-throughput methods may be an important parallel approach to drug-discovery and mechanistic research. The nematode worm *Caenorhabditis elegans* is an ideal candidate model. A rich subset of its behaviour can be studied, and hundreds of behavioural features can be quantified, then aggregated to yield a ‘signature’. Perturbation of this behavioural signature may provide a tool that can be used to quantify the effects of anaesthetic regimes, and act as an outcome marker for drug screening and molecular target research. Larval *C. elegans* were exposed to: isoflurane, ketamine, morphine, dexmedetomidine, and lithium (and combinations). Behaviour was recorded, and videos analysed with automated algorithms to extract behavioural features. Anaesthetic exposure during early development leads to persisting behavioural variation (in total, 125 features across exposure combinations). Higher concentrations, and combinations of isoflurane with ketamine, lead to persistent change in a greater number of features. Morphine and dexmedetomidine do not appear to lead to behavioural impairment. Lithium rescues the neurotoxic phenotype produced by isoflurane. Findings correlate well with vertebrate research: impairment is dependent on agent, is concentration-specific, is more likely with combination therapies, and can potentially be rescued by lithium. These results suggest that *C. elegans* may be an appropriate model with which to pursue phenotypic screens for drugs that mitigate the neurobehavioural impairment. Some possibilities are suggested for how high-throughput platforms might be organised in service of this field.

## Introduction

It is increasingly apparent that general anaesthetic agents can affect neurodevelopmental processes other than consciousness, and that these effects can persist beyond the period of clinical anaesthesia^1^. In fact, if we consider their presumptive mechanisms of action, it seems optimistic that we ever thought anaesthetics should have only short-term and reversible actions on consciousness. They appear to act promiscuously on receptor targets such as γ-Aminobutyric acid (GABA), N-methyl-d-aspartate (NMDA), and a variety of other protein channels, and are typically given at large concentrations to overcome their lack of selectivity^2^. These targets are widespread, and have defining roles to play in neural development and plasticity^3–5^. It is unsurprising therefore that interruption of consciousness at particular timepoints in brain age may be accompanied by extra-modal effects on the neuronal architecture, network or function.

Although there is genuine potential to harness these effects for good, (e.g. the modulation of glutamergic signalling by ketamine in the treatment of depression^6^), there is also significant public health concern^7^. Research in animals has demonstrated apoptotic neurodegeneration in the developing brain and persistent impairments in learning and memory, associated with exposure to volatile anaesthetics, ketamine, nitrous oxide, midazolam and propofol^8–10^. In general, longer exposures and combinations of anaesthetics delivered together have resulted in the most striking phenotypes. This phenomenon has been termed anaesthesia-induced neurotoxicity (AIN), and the findings have been replicated in multiple different organisms, including non-human primates^11^. Ethical and practical constraints mean that trials in human children have found it difficult to separate the role of anaesthesia from those of surgery, co-morbidity, socio-economic factors and other confounders^12^. Thus a mixed picture has emerged from clinical studies^13–17^. Animal models then, have an important role to play, particularly in elucidating mechanism, developmental windows of vulnerability, effects on neural architecture, and ability to quantify and therapeutically modulate the response.

This last consideration is the motivation for this work: how to apply rigorous quantitative analysis to record neurodevelopmental outcomes after exposure to anaesthesia, in a way which allows rapid and scalable screening of agents and modulating factors? Although mammalian models have provided much insight, there are drawbacks: vertebrates exhibit many degrees of freedom in their behaviour but only a limited number of heterogeneous outcomes have been used to quantify responses to anaesthesia exposure, leading to constrained expressions of nervous system output. Different groups have used, variously, open field tests^18^, fear conditioning tests^19^, assorted tests of spatial awareness, learning and memory^20,21^, and tests which were initially developed to assess depressant behaviour such as the forced swimming test^22^. The lack of standardization limits the power of both individual studies and meta-analyses, and in the absence of a generally accepted behavioural phenotype for AIN, it is difficult to be sure whether the constrained set of behaviours exhibited in these experiments can truly reflect the neuropathological deficit.

**Figure 1:**
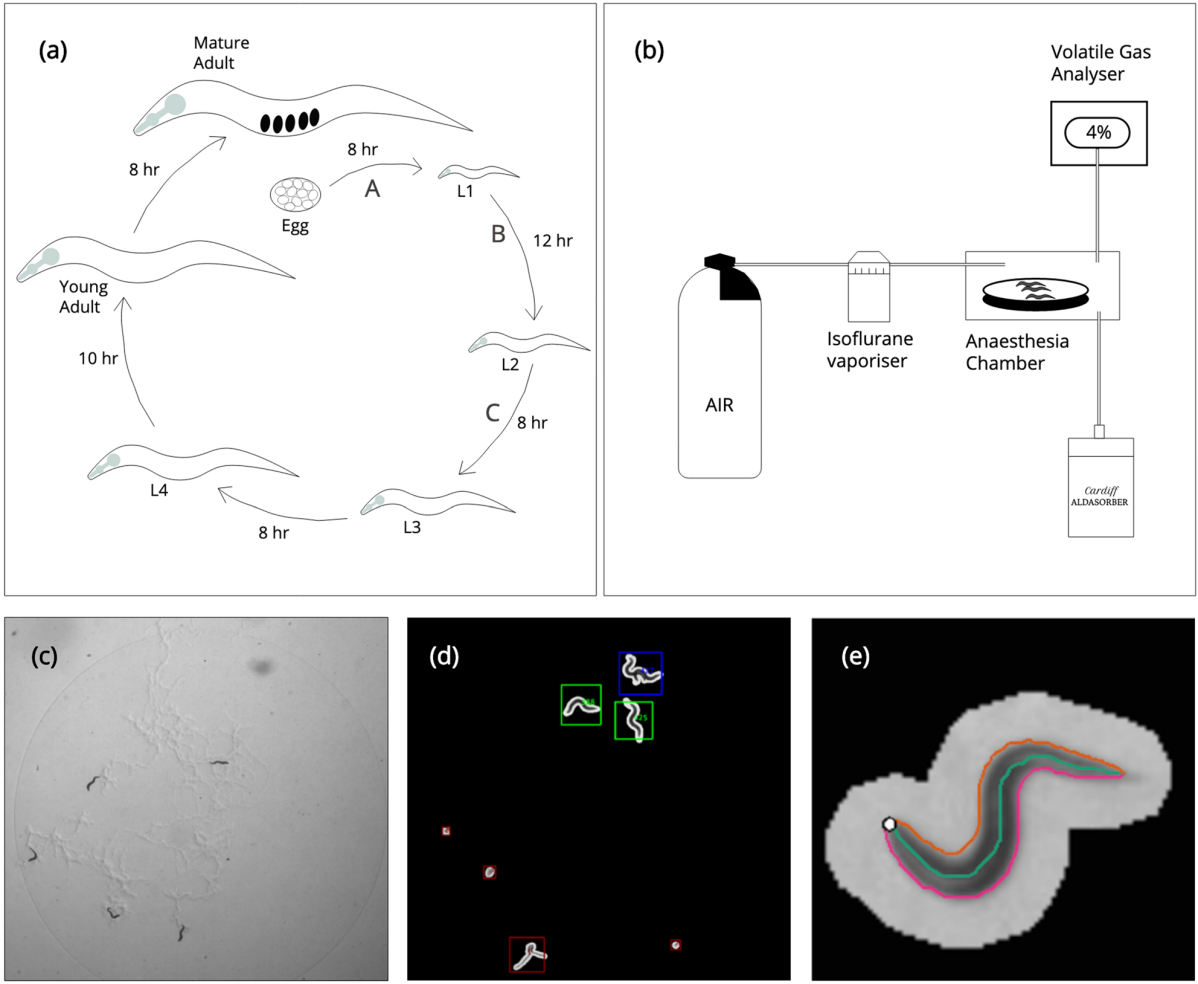
Experimental setup and worm tracking. (**a**) Life cycle of *C. elegans* hermaphrodite at 22 °C from egg to mature adult via larval stages. Capital letters refer to periods of neurogenesis: A, during proliferation phase of embryogenesis leading to 222 neurons at hatching; B, 74 neurons added during L1; C, 6 neurons added during L2. (**b**) Anaesthesia apparatus. Air flows from a pressurised cylinder into a vapouriser, and is saturated with a variable concentration of the volatile gas anaesthetic isoflurane. It then passes into a chamber holding plates of *C. elegans*. The delivered concentration is measured directly with an optical analyser, and titrated to desired value. The output from the chamber flows into the Cardiff Aldasorber, which binds anaesthetic gases for safe disposal. (**c**) Video still of experimental plate, showing 5 L1 larvae on an *E. coli* OP50 lawn. (**d**,**e**) Larva segmented from background using intensity threshold to extract the worm contour and skeleton.

For these reasons, until there is better mechanistic understanding of AIN, a high-throughput phenotypic screening method may be an important parallel line of investigation. This approach is difficult in mammals, but has been successfully demonstrated with other whole-animal models, in particularly the nematode worm *Caenorhabditis elegans*^23,24^. Because of its small size, short life cycle (Fig. 1a), and ease of maintenance and propagation, *C. elegans* can be raised in large numbers in the lab. Key anaesthetic targets including GABA and NMDA receptors are conserved in *C. elegans* and neuronal cells in the worm undergo apoptosis through conserved mechanisms first discovered in *C. elegans*^25^. Larval worms have previously been shown to develop neuronal and behavioural changes after exposure to isoflurane anaesthesia^26–28^. However, the potential of *C. elegans* goes further: its behaviours are amenable to quantitative aggregation and analysis, which yields a phenotypic ‘signature’. Perturbation of this behavioural signature in response to anaesthesia exposure may provide a powerful tool that can be used to quantify the effects of varied anaesthetic regimes, and act as an outcome marker for drug screening.

There are numerous theories linking anaesthesia exposure to neurodegeneration and behavioural impairment^29,30^. These include: disruptions in neuronal activity, signalling and neurotransmitter function; impaired neuronal maturation, integration, dendritic spine and synapse formation; abnormalities in growth and nutrient signalling pathways; microglia-related mechanisms; oxidative stress; impairment of autophagy; and epigenetic regulation. In *C. elegans* there is evidence to suggest that isoflurane may cause the neurotoxic effect by pathological activation of stress-response pathways. These effects have been shown to be blocked by of loss-of-function mutations in the insulin-like growth factor receptor gene *daf-2*^27^, the FoxO transcription factor *daf-16*^28^, and rapamycin-mediated inhibition of the mechanistic Target of Rapamycin (mTOR)^27,28^, consistent with the stress pathway hypothesis. However, no single theory unifies these mechanisms, or accounts for all reported histopathological and neurobehavioral phenotypes. Given the range of possible mechanisms as well as the uncertainty around each of them, a phenotypic screening approach can generate both candidate drugs for further testing as well as new mechanistic hypotheses. In this work, we generated a behavioural dataset resulting from the exposure of a developing nervous system to anaesthesia, using a whole animal *C. elegans* model. We provide proof of concept that a *C.* elegans-based high-throughput phenotypic screening system can aid in drug discovery and mechanistic research in AIN.

## Methods

The N2 strain of *C. elegans* was used in all experiments. Worms were maintained as per previously published protocols^31,32^. Larvae were synchronised to a 1-h time window as follows. 20 well-fed adult worms were left to lay eggs overnight. In the morning, all worms were washed off the plate using M9 buffer, leaving only eggs. Plates were left for 1 h, and then inspected. Any larvae that had hatched in this 1-h period were retrieved by washing the plate three times with 0.5 ml M9 buffer and centrifuging the larvae-containing liquid at 2500 rpm for 30 s. The supernatant was discarded, and the larvae transferred to a new 35 mm low-peptone plate pre-seeded with 10 μl *E. coli* OP50 bacteria (The protocol for preparing these plates is found here: https://dx.doi.org/10.17504/protocols.io.2rcgd2w). Typically, this step would yield several dozen animals. At all times other than during video recording, plates were kept in a 22 °C incubator. At 1 h after being transferred to the tracking plate (i.e. 1–2 h after hatching), larvae were exposed to anaesthesia. This timepoint was chosen because it is a period of extensive neurogenesis and neural rewiring (Fig. 1a); exposure to isoflurane at this stage has previously been showed to result in defective behaviour^26,28^. Agents were chosen because of previous vertebrate literature suggesting a neurotoxic effect (ketamine^8^, isoflurane^9^), lack of effect (morphine^33^, dexmedetomidine^34^) or potential to rescue the neurotoxic deficit (lithium^35^).

### Anaesthetic exposure and video recordings of behaviour

#### Isoflurane

Larvae were exposed at concentrations of 0% (control), 1%, 2% or 4% in air (flow rate 0.3 L/min). 4% has previously been shown to be the concentration at which neuronal activity is disrupted and physical quiescence is established^36^. The apparatus for delivering isoflurane is shown in Fig. 1b. Exposure was for 2 h, and animals were then transferred to new plates to emerge from anaesthesia. Control animals were exposed to air-flow only within the anaesthesia chamber. Videos of larval behaviour (see Fig. 1c for video still) were recorded for 15 min at the following intervals after hatching: (i) 8–9 h, corresponding to late L1 stage; (ii) 23–24 h, corresponding to mid-L3; (iii) 3 h after eggs seen on control plate (corresponding to adult). Of note, animals therefore had 5 h to recover from exposure before the first video, and isoflurane is expected to have cleared fully in < 1 h. 4–6 larvae were recorded per plate, and 3 plates were exposed at each concentration. The videos were recorded using a custom-made multi-worm tracker built by Real Time Computing (East Sussex, UK).

#### Other agents

Synchronised larvae were transferred onto a plate containing a liquid layer (added 1 h previously) of 1 µM, 10 µM or 100 µM of the following drugs: ketamine hydrochloride, morphine sulphate, dexmedetomidine hydrochloride (1 µM and 10 µM only—attempts to use 100 µM concentrations resulted in drug precipitation), and lithium chloride. These plates were created as follows: stock drug of 100 mM was made up in DMSO, and serial dilutions made to 10 mM and 1 mM solutions. 3.5 μl of each was then added to 3.5 ml low-peptone NGM agar plates (i.e. 1:1000 dilution) to produce the desired concentrations. The following combinations were also studied: (a) Isoflurane 1% with Ketamine 1 µM, 10 µM and 100 µM; (b) Isoflurane 2% with Ketamine 1 µM, 10 µM and 100 µM; (c) Isoflurane 4% with Ketamine 1 µM, 10 µM and 100 µM; (d) Isoflurane 4% with Lithium 1 µM, 10 µM and 100 µM. Exposure was for 2 h and animals were then transferred to new plates. Post-exposure recording was as given above.

### Data analysis

Worms were tracked using the open source Tierpsy Tracker^37^. Briefly, worm pixels were segmented from the background using an intensity threshold, and the contour of each segmented object is extracted and given a unique identifier (Fig. 1d, and also supplementary Video S1). The worm skeleton is then defined as the midline connecting the two points of highest curvature (head and tail, Fig. 1e). The body of the worm is split into head, neck, midbody, hips and tail (and sub-divided further). Bend angles can be calculated as the difference in tangent angles at any given point. Combining this information with knowledge of time and the worm’s location on the plate allows extraction of a range of behavioural features, including parameters related to morphology, posture, locomotion and path dynamics^38^. Supplementary Figure S1, reprinted from Javer et al.^38^, illustrates the schematics of these core features. The full set runs to over a thousand features; however, a subset of 256 features were chosen, as analysis has previously shown that a similar subset provides a good balance between accurate classification and reducing the handicap associated with correction for multiplicity^38^. These are given in supplementary data Table S1. Data was filtered so that only worm identities which existed for a minimum of 30 s were accepted, to minimize the effect of artefacts and segmentation errors which occasionally give rise to short-lived non-worm identifiers. Unpaired Student’s t-test was used to compare feature means between groups and we controlled the false discovery rate using the Benjamini-Yekutieli procedure^39^.

Data was analysed using MATLAB (R2016a, Mathworks, Massachusetts, USA).

## Results

### Administration of anaesthetics during early development has no gross effects on growth, as measured by adult length

**Figure 2:**
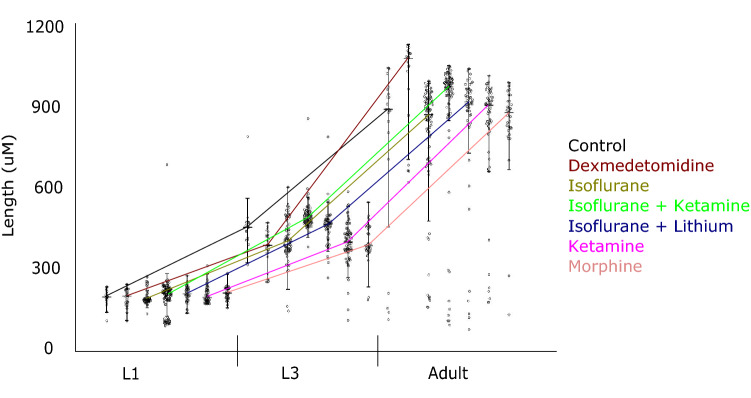
Early administration of anaesthetics has no gross effects on growth but can have long term effects on behaviour. (**a**) Lengths at late-L1, mid-L3, and adult stages. Results for different drug concentrations are pooled. Boxplots given distribution of lengths, with individual data points overlain (each point is the average value for one worm trajectory). Coloured lines represent experimental condition, and connect median lengths at each stage. Growth is as expected, and there are no significant differences in adult size. Analysis of variation (ANOVA) of adult lengths supports the null hypothesis, that population means are equal (F 1.44 < Fcrit 1.56).

Analysis of variation (ANOVA) of adult lengths in animals exposed to each different drug concentration supports the null hypothesis, that population means are equal (F 1.44 < Fcrit 1.56). Figure 2 displays worm lengths, pooled across the different drug concentrations for easy of display, for control and anaesthetic experiments. Boxplots give the distribution of lengths, with individual data points overlain. Coloured lines connect the median length at each developmental stage. Growth is as expected, and all animals follow similar trajectories. Other size features, e.g. area, show a similar pattern (data not shown).

### There are developmental stage- and exposure-related variations in behaviour which persist long after emergence from anaesthesia

**Figure 3:**
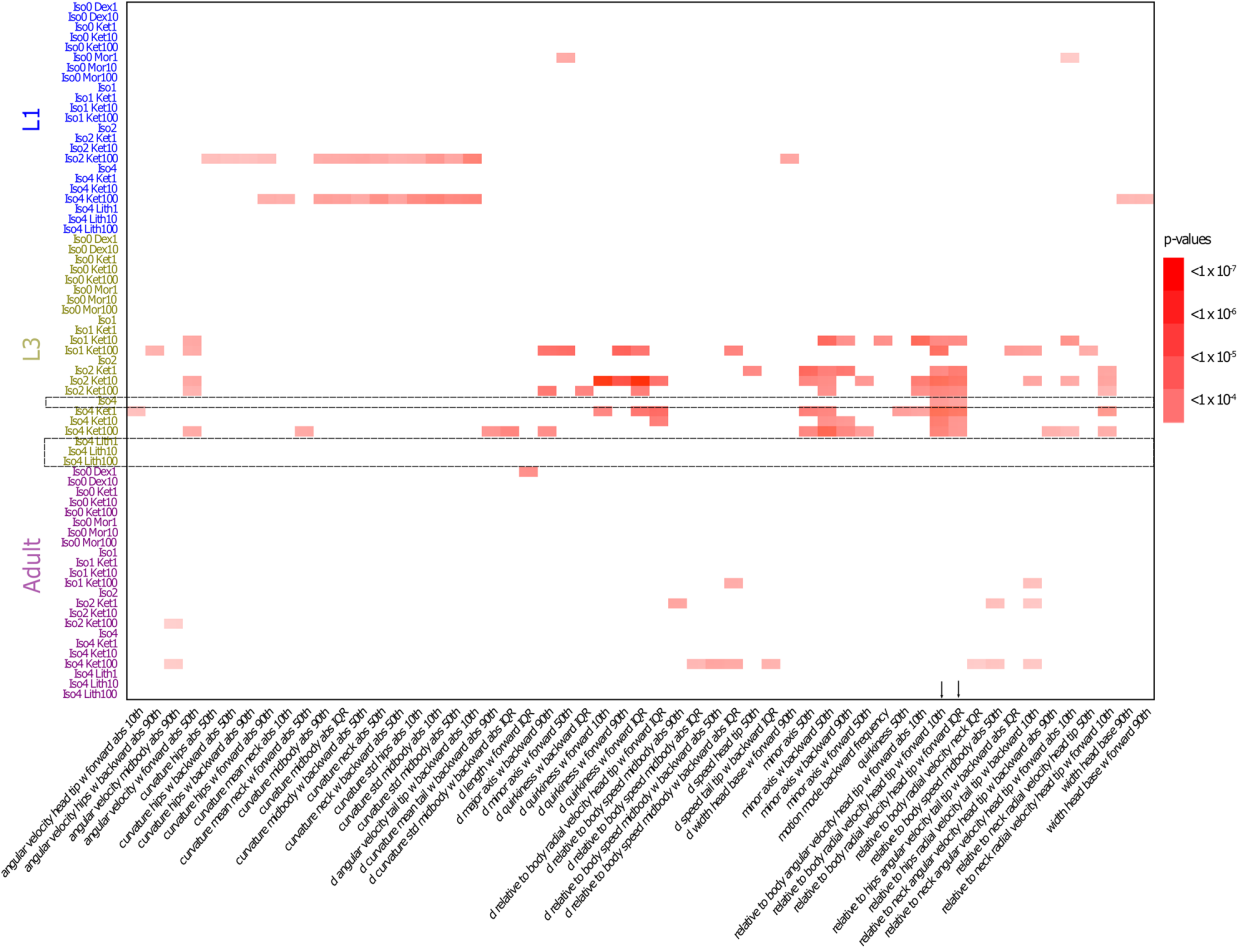
Heatmap shows p-values of anaesthetic condition/behavioural feature combinations that were found to be significantly different to control after multiplicity correction. Experimental conditions are listed on the y axis, arranged by developmental stage. Dex = dexmedetomidine, Ket = ketamine, Mor = morphine, Iso = isoflurane; Lit = lithium. Numbers refer to either isoflurane concentration in percent air, or drug concentration in μM. Behavioural features are listed on the x axis. Significant condition/feature combinations are represented by red rectangles, with p-value ranges as indicated (white indicates no significant difference). The phenotype of animals exposed to certain anaesthetic conditions varies from control, and this variation is developmental-stage-specific—changes seen at one stage are not necessarily carried over to a later stage, and new changes can manifest as the animal develops. Dashed rectangles and arrows highlight two hits at L3 after exposure to Isoflurane 4%; in each case, the difference from control is abrogated after co-administration with lithium (see also Fig. 5).

Figure 3 is a heat map of features that were found to be significantly different between anaesthesia-exposed animals and controls after correction for multiple comparisons. The data shows that the behavioural phenotype of animals exposed to certain anaesthetic conditions, but not others, varies from that of control animals. This variation is developmental-stage-specific, i.e. variations in phenotype seen at one stage of larval development are not necessarily carried over to a later stage, and new variations can manifest as the animal develops.

In total, there are 29 instances of a significant exposure/feature effect at L1, 81 at L3, and 15 at adult. Single-agent exposure with isoflurane yields 2 significant ‘hits’, both at L3 and at the highest 4% concentration only. There are no hits with single-agent exposure to ketamine at any concentration. However, the combination of isoflurane and ketamine leads to many persisting behavioural changes. There is evidence of a concentration effect—with exposure to higher concentrations causing more feature differences. The isoflurane 4% + ketamine 100 µM combination leads to 13 feature hits at L1, 14 at L3, and 8 at adult. Other concentrations of the isoflurane + ketamine combination also show high numbers of feature hits. There are only 2 hits for morphine (at 1 µM but not higher concentrations), and a single hit for dexmedetomidine (at 1 µM but not higher) in total.

**Figure 4:**
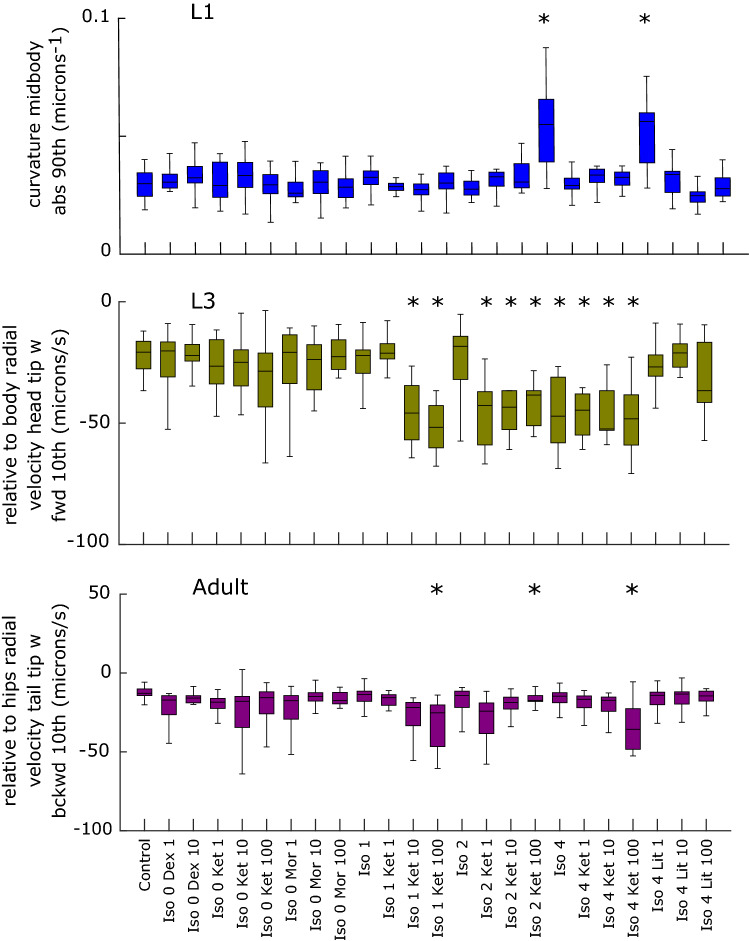
Examples of significant anaesthetic condition/behavioural feature differences at L1, L3 and adult. Combinations significantly different to control are marked with an asterix (p value limits as given in Fig. 3). Dex = dexmedetomidine, Ket = ketamine, Mor = morphine, Iso = isoflurane; Lit = lithium. Numbers refer to either isoflurane concentration in percent air, or drug concentration in μM.

Figure 4 displays boxplots showing examples of individual feature differences at L1 (top panel), L3 (middle) and adult (bottom) stages. The latter findings show that behavioural changes persist after the period of neurogenesis, which is expected to be completed by L3.

### Co-administration of lithium rescues the behavioural effects of isoflurane administration

**Figure 5:**
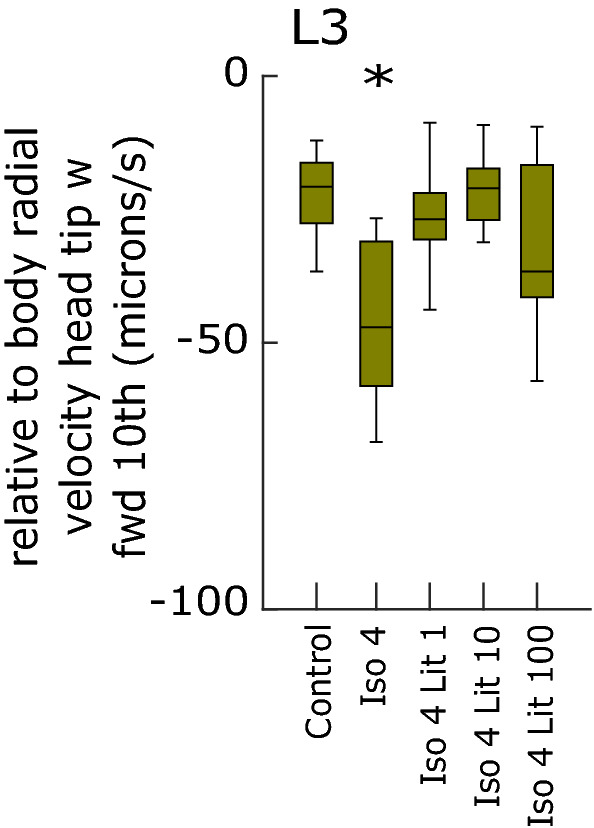
Boxplot depicting one example of a feature that is significantly different at L3 stage after administration of Isoflurane 4% alone. Co-administration of lithium abrogates the difference with controls. Iso = isoflurane; Lit = lithium. Numbers refer to either isoflurane concentration in percent air, or drug concentration in μM.

In this dataset, there are two feature hits resulting from exposure of larvae to Isoflurane 4% alone. Both are seen at L3 stage. In each case, co-administration of lithium at concentrations of 1, 10 or 100 µM abrogated the differences with controls, without leading to any new difference in other features. Dashed rectangles in Fig. 3 highlight the relevant experimental conditions (Isoflurane 4%, and Isoflurane 4% co-administered with Lithium 1 µM, 10 µM and 100 µM respectively); arrows point to the two feature examples where significant differences are seen with Isoflurane 4%, but abrogated when lithium is co-administered (Fig. 3). Figure 5 is a boxplot representing one of these features (relative to body radial velocity head tip w forward 10th).

## Discussion

We find that *C. elegans* larvae exposed to anaesthesia shortly after hatching display later behavioural differences when compared to controls. This is consistent with results from previous studies which exposed *C. elegans* to isoflurane alone^27,28^. Our research further establishes that the effect is agent- and concentration-dependent, with combinations of isoflurane and ketamine at higher doses consistently being correlated with significant differences. In this, the effect is similar to that seen in vertebrates, where the most robust and consistent findings of neurobehavioural impairment also come in experiments with multiple agents and stronger exposures^9,40,41^. We have also seen that morphine and dexmedetomidine, both agents which have been shown not to cause neuroapoptosis or behavioural change in vertebrates^33,34^, do not appear to cause lasting behavioural change in *C. elegans*. Although there are 2 hits for morphine and 1 for dexmedetomidine, the pattern of these, with hits in lower concentrations not being reproduced for higher concentrations, suggests that they are outliers.

There is some evidence here that lithium, which has been shown to rescue the neurotoxic phenotype in vertebrates^35^, may have a similar effect in *C. elegans*. Table 1 summarises the parallels between results from these experiments and vertebrate findings. The fact that results in the worm are broadly consistent with those in vertebrates suggests that these experiments could form the basis of a high-throughput screening system to detect compounds which could modulate anaesthesia-induced neurobehavioural impairment, and be used to identify mechanisms and genes involved in the phenomenon.

**Table 1:** Summary of evidence: anaesthetic exposure to *C. elegans* in these experiments, and previous literature from vertebrate experiments.

Anaesthetic conditions	*C. elegans* results in these experiments	Vertebrate findings^9,33,35,40,41,55–57^
Isoflurane	Evidence of behavioural impairment after exposure to 4% isoflurane as early L1, detectable at L3 (but not at late L1, and not persisting to adult)	Multiple studies from rodent and other animals, including non-human primates, provide evidence of histopathological changes and behavioural impairment
Ketamine	Non-significant results in these experiments from single-agent exposure	Evidence of histopathological change and behavioural impairment
Morphine	No evidence of persisting behavioural change	No evidence of histopathological or behavioural change
Dexmedetomidine	No evidence of persisting behavioural change	No evidence of histopathological or behavioural change
Concentration effect	Isoflurane at 4% is associated with some behavioural feature changes at L3. Lower concentrations are not	Higher dose-exposure parameters lead to increased histopathological and behavioural change
Multi-drug cocktails	Ketamine/Isoflurane combination produces strongest evidence of persisting behavioural change	Exposure to multi-drug cocktails (including combinations of volatile anaesthetics, ketamine, and benzodiazepines), lead to pronounced histopathological and behavioural change
Lithium	Rescues behavioural effect seen with Isoflurane 4%	Evidence for rescuing isoflurane-induced neuroapoptosis in the infant primate brain

Many genes (around 80% by some estimates) and molecular pathways are known to be conserved between *C. elegans* and vertebrates^42,43^, and the worm has been used extensively for drug discovery and target identification. Forward genetic screens, which start with a phenotype of interest and aim to identify the genetic basis for the behaviour, have identified genes and products relevant to human disease phenotypes, including in neurodegenerative and neuromuscular disorders^44,45^. Chemistry-to-gene screens, in which mutagenized animals are exposed to a drug of interest and then screened for resistance to drug effect, have helped uncover clinically important molecular targets, such as those for antiparasitic drugs^46^. Gene-to-chemistry screens can facilitate the discovery of new drug targets by silencing genes in the search for resistance to a compound of interest. This approach led to the discovery that one compound in a library reduced muscular degeneration in a nematode model of Duchenne muscular dystrophy (DMD)^47^. The drug was the steroid prednisolone, in clinical use in DMD to palliate symptoms, and providing proof of principle of the potential of *C. elegans* to identify compounds for use in humans.

The advance of our mechanistic understanding of anaesthesia-induced neurobehavioural impairment, and the discovery of new compounds to ameliorate the phenomenon have both been recognised as research priorities. Existing methods have thus far produced an incomplete picture, and led to the identification of very few candidate drugs, most of which are years away from trials. It is possible to see how *C. elegans* could be used as an inexpensive, quick and tractable model for screening, with the aim of filtering out compounds or targets of interest for further validation. In contrast to cell line screening, whole-organism behavioural screens allow for unbiased investigation of a broad range of disease-related pathways, not just a cellular marker that is decided upon a priori*.* Given the gaps in our knowledge of the pathway towards behavioural impairment, this is an advantage.

Some approaches suggest themselves: the first is drug-discovery using libraries of pre-approved or novel compounds. These are unlikely to reveal new anaesthetic agents clearly, but may produce hits which enhance or supress the phenotype. Given the results seen in these experiments, a good starting point may be to expose worms as L1s to an isoflurane/ketamine/experimental drug combination, and measure for effect at L3, the stage at which the greatest perturbation of the phenotypic signature was recorded. We could speculate as to why L3 is stage at which behavioural impairment is most marked; perhaps the pertinent neural pathways are not fully developed by late-L1, and perhaps compensatory mechanisms have set in by adulthood despite the fact that neurogenesis is complete.

Further, mutagenesis of wildtype *C. elegans* may reveal mutants in whom the phenotype is enhanced or supressed, and provide a starting point for investigating molecular targets involved in the pathway. Previous work in this vein with isoflurane-exposed *C. elegans* has implicated *daf-2* and *daf-16* pathways, (and the mechanistic Target of Rapamycin (mTOR), which interacts with these)^27,28^. These pathways regulate stress-response. Taken together, the findings suggest that activation of neuronal stress response pathways may be involved in the persistent behavioural changes seen in *C. elegans* after exposure to Isoflurane. mTOR also negatively influences autophagy, another posited mechanism for AIN^48^. Interestingly, lithium has been shown to enhance autophagy via an mTOR-independent pathway^49^, providing mechanistic support for its ability to rescue the neurotoxic phenotype.

A complementary approach would be to employ a reverse genetic screen, using genome-wide RNAi libraries to systematically silence genes in the search for a mutant which is resistant to the compounds of interest—either the isoflurane/ketamine combination itself, or a drug which rescues the phenotype. With automated methods of dispensing worms and assay components, and new technologies for assay readouts and image analysis, such screens have significant potential to detect compounds and mechanisms of action which could have proven elusive to vertebrate research.

There are some limitations to this work. It is difficult to compare bioactivity in the nematode worm with human equivalents, because of the presence of the cuticle, uncertainty about pharmacokinetics and pharmacodynamics, and the effect of temperature on the potency of volatile anaesthetics. However, bioactivity has previously been established in *C. elegans* for all the compounds used in this work^28,50–52^ with the exception of dexmedetomidine. The latter is a centrally-acting α_2_ adrenoreceptor agonist in vertebrates. There is no adrenoreceptor system in *C. elegans*; however a variety of endogenous amine neurotransmitters play a role in the regulation of a diverse set of behaviours^53^, by activating G protein-coupled receptors that share some similarities with vertebrate adrenoreceptors.

Only isoflurane/ketamine combinations show strong and persisting effects on behavioural change. This is broadly in keeping with vertebrate findings, although some groups have demonstrated behavioural change with single-agent exposure alone (e.g. Lin et al.^18^). It is possible that other *C. elegans* based screening protocols may be more sensitive to single-agent exposure, though this may not necessarily be an advantage if single-agent exposure does not lead to human impairment (as the best current evidence suggests^17^). In addition, although we have some evidence here that co-administration of lithium can abolish the behavioural effect seen with isoflurane exposure, it would be instructive to establish whether this rescue applies after treatment with combination exposures.

The chosen methods of phenotyping focus on features which are amenable to automated analysis, rather than high-order behaviours such as chemotaxis, which have previously been shown to be deficient after anaesthesia exposure^26^. However, this also allows unbiased screens, rather than making a priori judgements about which behaviours are likely to be mechanistically linked to the neuropathology induced by anaesthesia exposure.

This approach allows interrogation of phenotypic aspects of anaesthesia-induced neurobehavioural impairment, in the service of drug discovery and target identification. The use of quantitative phenotyping greatly enhances the power to detect ‘hits’—compounds, genes and pathways of interest—over manual observation alone. These hits can then be further validated in vertebrate models or extended screens. Developments in robotics, microfluidics and image-analysis now allow for true high-throughput screening of nematodes^23,24,54^. *C. elegans* therefore is an excellent model with which to pursue drug discovery and mechanistic research. This work provides proof of principle for its use as a low-cost, high-throughput, whole-animal screening tool in the search for novel compounds and molecular mechanisms in AIN.

## Supplementary Information

Supplementary Figure S1.

Supplementary Table S1.

Supplementary Video S1.

## Data Availability

The datasets generated during this study are available: https://doi.org/10.5281/zenodo.4725145.
