# Supplementary material for: Quantitative behavioural phenotyping to investigate anaesthesia induced neurobehavioural impairment
Source: Sci Rep. 2021 Sep 29;11:19398. doi: 10.1038/s41598-021-98405-x (PMC8481492; doi:10.1038/s41598-021-98405-x)
Supplement: Supplementary file 1 — Supplementary Figure S1. [file 41598_2021_98405_MOESM1_ESM.pptx]

## Slide 1
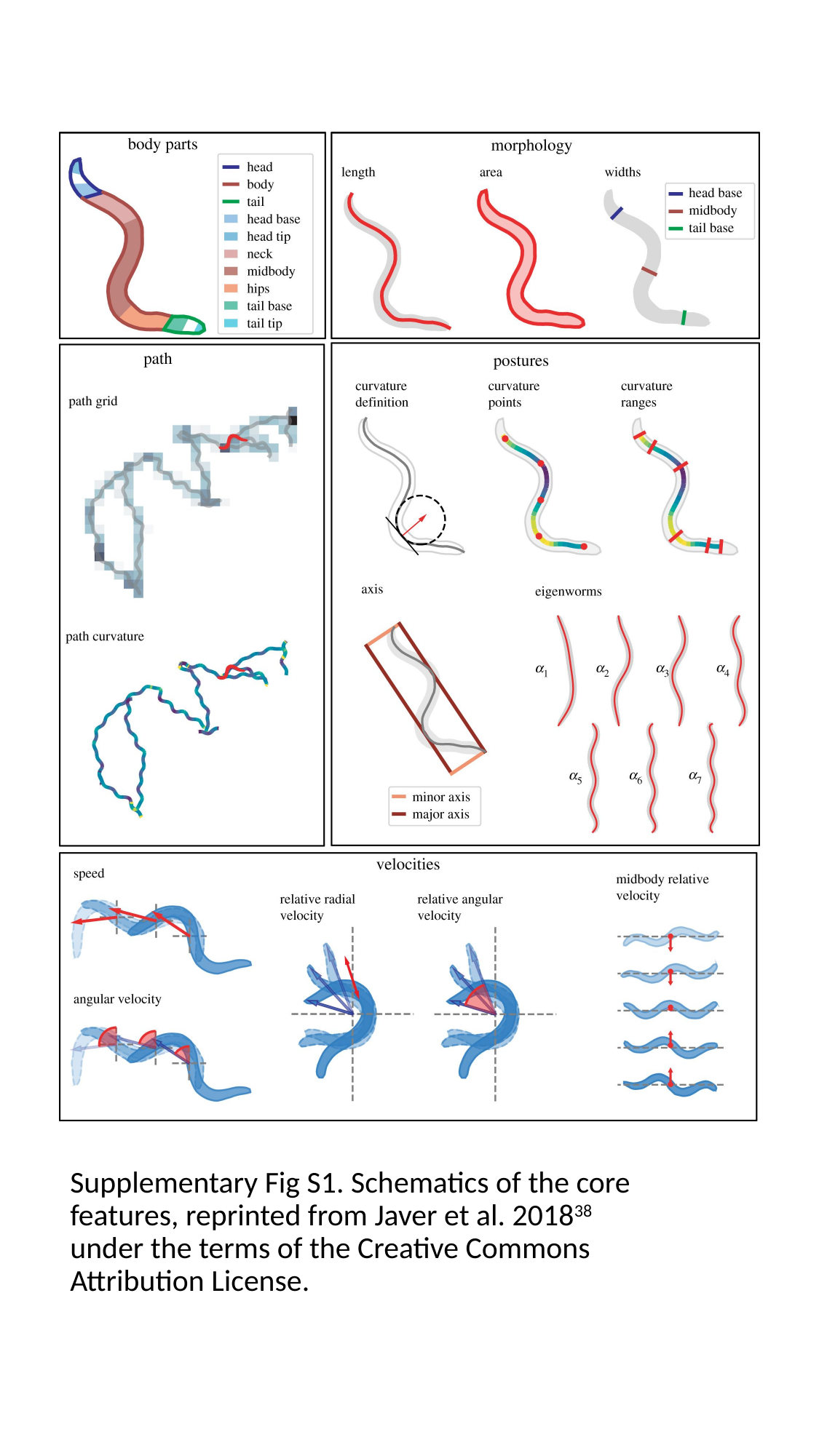

Supplementary Fig S1. Schematics of the core features, reprinted from Javer et al. 201838 under the terms of the Creative Commons Attribution License.
