# Supplementary material for: Quantitative behavioural phenotyping to investigate anaesthesia induced neurobehavioural impairment
Source: Sci Rep. 2021 Sep 29;11:19398. doi: 10.1038/s41598-021-98405-x (PMC8481492; doi:10.1038/s41598-021-98405-x)
Supplement: Supplementary file 2 — Supplementary Table S1. [file 41598_2021_98405_MOESM2_ESM.docx]

## Supplementary Data

Table S1. ‘Tierpsy_256’ features^35^. There are many sets of 256 features which perform similarly in classification. The following is one such set, used in this analysis.

| relative_to_head_base_radial_velocity_head_tip_w_forward_10th |
| --- |
| d_relative_to_head_base_radial_velocity_head_tip_w_forward_90th |
| d_relative_to_head_base_angular_velocity_head_tip_abs_50th |
| motion_mode_backward_fraction |
| angular_velocity_tail_tip_w_backward_abs_IQR |
| curvature_std_midbody_abs_10th |
| motion_mode_paused_frequency |
| d_curvature_mean_tail_w_backward_abs_90th |
| curvature_std_neck_w_backward_abs_90th |
| major_axis_w_forward_10th |
| d_relative_to_body_radial_velocity_head_tip_w_forward_IQR |
| d_area_w_backward_10th |
| curvature_std_tail_abs_90th |
| curvature_mean_head_abs_90th |
| curvature_mean_head_w_backward_abs_90th |
| relative_to_head_base_radial_velocity_head_tip_50th |
| angular_velocity_hips_w_forward_abs_10th |
| relative_to_hips_radial_velocity_tail_tip_w_backward_90th |
| d_major_axis_50th |
| width_tail_base_w_backward_10th |
| d_relative_to_head_base_angular_velocity_head_tip_w_forward_abs_90th |
| curvature_tail_w_forward_abs_90th |
| relative_to_head_base_angular_velocity_head_tip_w_forward_abs_IQR |
| curvature_head_w_forward_abs_IQR |
| width_midbody_10th |
| d_width_head_base_10th |
| d_relative_to_body_speed_midbody_w_forward_abs_90th |
| d_quirkiness_IQR |
| d_angular_velocity_tail_base_abs_50th |
| width_midbody_w_forward_10th |
| curvature_std_head_w_forward_abs_50th |
| relative_to_neck_radial_velocity_head_tip_50th |
| relative_to_head_base_radial_velocity_head_tip_w_forward_IQR |
| length_IQR |
| d_angular_velocity_head_tip_abs_10th |
| curvature_head_abs_10th |
| minor_axis_w_forward_10th |
| d_length_IQR |
| path_density_midbody_95th |
| relative_to_neck_angular_velocity_head_tip_w_forward_abs_50th |
| d_curvature_neck_w_forward_abs_IQR |
| relative_to_head_base_radial_velocity_head_tip_w_forward_90th |
| angular_velocity_midbody_abs_90th |
| curvature_neck_abs_50th |
| relative_to_body_radial_velocity_tail_tip_w_backward_10th |
| d_curvature_midbody_w_backward_abs_10th |
| d_angular_velocity_head_tip_w_forward_abs_50th |
| minor_axis_w_backward_50th |
| relative_to_hips_radial_velocity_tail_tip_w_backward_10th |
| width_head_base_w_forward_IQR |
| d_length_50th |
| d_speed_head_tip_w_forward_90th |
| curvature_mean_tail_w_backward_abs_50th |
| path_coverage_tail |
| relative_to_body_radial_velocity_hips_w_forward_90th |
| curvature_std_hips_abs_10th |
| curvature_std_head_w_backward_abs_10th |
| relative_to_neck_radial_velocity_head_tip_w_forward_50th |
| width_head_base_w_forward_50th |
| minor_axis_50th |
| width_head_base_w_forward_10th |
| d_quirkiness_w_forward_IQR |
| d_area_10th |
| width_head_base_50th |
| relative_to_head_base_angular_velocity_head_tip_w_forward_abs_90th |
| curvature_std_neck_abs_IQR |
| motion_mode_backward_frequency |
| curvature_std_neck_abs_10th |
| d_width_midbody_w_backward_90th |
| d_relative_to_head_base_angular_velocity_head_tip_w_backward_abs_50th |
| curvature_std_hips_w_forward_abs_90th |
| curvature_std_neck_w_forward_abs_10th |
| curvature_mean_neck_w_forward_abs_90th |
| d_curvature_std_midbody_w_backward_abs_IQR |
| angular_velocity_tail_tip_w_backward_abs_90th |
| minor_axis_w_backward_90th |
| d_relative_to_body_radial_velocity_head_tip_50th |
| curvature_mean_head_w_forward_abs_50th |
| curvature_mean_hips_w_forward_abs_50th |
| d_width_head_base_w_forward_90th |
| quirkiness_w_forward_90th |
| curvature_mean_neck_w_forward_abs_IQR |
| quirkiness_50th |
| curvature_hips_w_backward_abs_10th |
| turn_intra_duration_50th |
| minor_axis_w_forward_50th |
| d_angular_velocity_head_tip_w_backward_abs_90th |
| relative_to_head_base_angular_velocity_head_tip_abs_50th |
| relative_to_neck_angular_velocity_head_tip_w_forward_abs_90th |
| d_minor_axis_w_forward_10th |
| d_angular_velocity_head_tip_w_backward_abs_IQR |
| curvature_head_abs_90th |
| d_angular_velocity_tail_tip_w_backward_abs_IQR |
| d_path_curvature_midbody_abs_90th |
| curvature_std_head_abs_IQR |
| curvature_mean_neck_abs_90th |
| d_minor_axis_w_forward_50th |
| relative_to_body_radial_velocity_tail_tip_w_forward_90th |
| curvature_std_neck_w_forward_abs_50th |
| curvature_std_neck_abs_90th |
| d_width_head_base_w_backward_90th |
| length_w_forward_IQR |
| relative_to_tail_base_angular_velocity_tail_tip_w_forward_abs_90th |
| d_relative_to_neck_angular_velocity_head_tip_abs_50th |
| d_quirkiness_10th |
| curvature_hips_w_backward_abs_50th |
| d_length_w_forward_IQR |
| d_width_head_base_w_backward_10th |
| d_curvature_std_head_abs_10th |
| relative_to_body_angular_velocity_tail_tip_w_backward_abs_IQR |
| curvature_tail_w_forward_abs_50th |
| d_curvature_std_hips_w_forward_abs_90th |
| quirkiness_w_forward_50th |
| quirkiness_w_backward_50th |
| motion_mode_backward_duration_50th |
| motion_mode_forward_frequency |
| curvature_std_midbody_w_forward_abs_IQR |
| relative_to_head_base_angular_velocity_head_tip_abs_IQR |
| relative_to_neck_radial_velocity_head_tip_w_forward_10th |
| quirkiness_90th |
| curvature_std_neck_w_forward_abs_IQR |
| relative_to_head_base_angular_velocity_head_tip_w_backward_abs_IQR |
| relative_to_body_angular_velocity_head_tip_w_forward_abs_10th |
| d_relative_to_body_speed_midbody_abs_90th |
| curvature_neck_w_backward_abs_IQR |
| curvature_midbody_w_backward_abs_50th |
| d_quirkiness_w_backward_IQR |
| relative_to_head_base_angular_velocity_head_tip_abs_10th |
| path_curvature_head_abs_50th |
| angular_velocity_w_backward_abs_IQR |
| d_curvature_std_head_w_backward_abs_50th |
| width_tail_base_90th |
| angular_velocity_head_tip_w_forward_abs_10th |
| angular_velocity_abs_IQR |
| curvature_head_w_backward_abs_90th |
| d_curvature_mean_head_w_backward_abs_90th |
| d_area_w_backward_IQR |
| relative_to_neck_angular_velocity_head_tip_abs_10th |
| width_tail_base_w_backward_IQR |
| path_curvature_head_abs_10th |
| width_midbody_w_forward_50th |
| relative_to_tail_base_radial_velocity_tail_tip_w_backward_10th |
| speed_neck_10th |
| width_head_base_IQR |
| d_curvature_std_head_w_backward_abs_IQR |
| relative_to_head_base_angular_velocity_head_tip_w_backward_abs_90th |
| d_relative_to_head_base_angular_velocity_head_tip_w_forward_abs_IQR |
| relative_to_hips_radial_velocity_tail_tip_50th |
| d_speed_tail_tip_w_backward_IQR |
| curvature_mean_tail_w_backward_abs_IQR |
| curvature_mean_neck_abs_10th |
| d_major_axis_w_backward_90th |
| relative_to_body_radial_velocity_neck_w_forward_90th |
| relative_to_neck_angular_velocity_head_tip_w_forward_abs_10th |
| d_relative_to_body_speed_midbody_abs_IQR |
| curvature_std_tail_w_forward_abs_90th |
| d_speed_tail_tip_50th |
| d_angular_velocity_hips_abs_10th |
| curvature_hips_abs_50th |
| d_quirkiness_w_forward_10th |
| relative_to_body_radial_velocity_head_tip_w_forward_IQR |
| curvature_std_neck_abs_50th |
| curvature_midbody_abs_90th |
| curvature_neck_w_forward_abs_IQR |
| d_speed_head_tip_50th |
| d_curvature_std_head_w_forward_abs_90th |
| curvature_mean_midbody_w_backward_abs_IQR |
| curvature_std_tail_abs_10th |
| curvature_mean_hips_abs_90th |
| relative_to_tail_base_radial_velocity_tail_tip_50th |
| d_length_w_forward_10th |
| d_relative_to_body_radial_velocity_neck_50th |
| width_midbody_w_backward_10th |
| d_curvature_std_head_w_forward_abs_10th |
| d_speed_tail_tip_w_forward_IQR |
| d_curvature_midbody_w_forward_abs_90th |
| speed_10th |
| d_curvature_std_neck_w_forward_abs_90th |
| curvature_neck_w_backward_abs_50th |
| d_angular_velocity_head_tip_w_forward_abs_90th |
| quirkiness_IQR |
| relative_to_body_radial_velocity_hips_50th |
| d_quirkiness_w_backward_90th |
| relative_to_hips_angular_velocity_tail_tip_w_backward_abs_IQR |
| width_tail_base_w_backward_90th |
| d_width_tail_base_50th |
| width_tail_base_10th |
| d_angular_velocity_head_tip_w_forward_abs_IQR |
| relative_to_head_base_angular_velocity_head_tip_abs_90th |
| d_relative_to_head_base_angular_velocity_head_tip_w_backward_abs_10th |
| curvature_std_midbody_abs_50th |
| curvature_hips_w_backward_abs_90th |
| d_speed_head_tip_w_forward_50th |
| curvature_head_abs_50th |
| curvature_std_tail_abs_IQR |
| curvature_neck_abs_90th |
| curvature_mean_neck_w_forward_abs_50th |
| angular_velocity_w_forward_abs_50th |
| d_curvature_std_head_w_forward_abs_IQR |
| d_curvature_midbody_abs_50th |
| width_head_base_w_forward_90th |
| relative_to_body_radial_velocity_head_tip_w_forward_10th |
| d_curvature_std_head_w_forward_abs_50th |
| width_head_base_90th |
| curvature_midbody_abs_IQR |
| d_curvature_std_neck_w_backward_abs_90th |
| d_relative_to_body_speed_midbody_w_backward_abs_IQR |
| curvature_hips_w_backward_abs_IQR |
| d_relative_to_head_base_radial_velocity_head_tip_w_backward_50th |
| relative_to_body_radial_velocity_tail_tip_w_forward_IQR |
| curvature_hips_w_forward_abs_90th |
| speed_w_forward_50th |
| width_midbody_w_forward_90th |
| d_angular_velocity_tail_tip_abs_90th |
| d_speed_w_backward_50th |
| width_tail_base_w_backward_50th |
| angular_velocity_hips_w_backward_abs_90th |
| d_width_midbody_w_forward_50th |
| curvature_mean_neck_w_backward_abs_90th |
| width_midbody_90th |
| curvature_std_tail_w_forward_abs_50th |
| relative_to_neck_angular_velocity_head_tip_w_backward_abs_90th |
| d_curvature_std_head_abs_50th |
| speed_tail_tip_w_forward_90th |
| curvature_mean_hips_w_backward_abs_IQR |
| speed_midbody_w_forward_90th |
| d_curvature_mean_hips_w_forward_abs_10th |
| speed_tail_base_w_backward_90th |
| relative_to_body_speed_midbody_abs_50th |
| d_length_w_backward_IQR |
| relative_to_body_radial_velocity_hips_w_backward_10th |
| curvature_tail_w_forward_abs_IQR |
| width_tail_base_w_forward_50th |
| d_quirkiness_w_forward_90th |
| d_curvature_std_head_abs_90th |
| angular_velocity_head_tip_abs_10th |
| d_relative_to_neck_angular_velocity_head_tip_w_backward_abs_90th |
| d_curvature_std_tail_w_forward_abs_50th |
| relative_to_body_angular_velocity_tail_tip_w_forward_abs_10th |
| relative_to_tail_base_angular_velocity_tail_tip_w_backward_abs_90th |
| speed_neck_w_forward_90th |
| d_length_w_backward_10th |
| width_tail_base_50th |
| curvature_std_hips_w_backward_abs_IQR |
| d_relative_to_body_speed_midbody_w_backward_abs_50th |
| d_speed_tail_base_w_forward_IQR |
| d_angular_velocity_tail_tip_w_backward_abs_10th |
| curvature_mean_head_w_backward_abs_50th |
| relative_to_body_radial_velocity_neck_IQR |
| d_width_tail_base_w_backward_50th |
| d_angular_velocity_tail_tip_w_forward_abs_50th |
| curvature_mean_hips_w_backward_abs_90th |
| curvature_std_tail_w_backward_abs_10th |
| curvature_head_w_backward_abs_50th |
| length_w_backward_IQR |
| d_relative_to_neck_radial_velocity_head_tip_w_forward_50th |
