# Supplementary figures and images for: Quantitative behavioural phenotyping to investigate anaesthesia induced neurobehavioural impairment
Source: Sci Rep. 2021 Sep 29;11:19398. doi: 10.1038/s41598-021-98405-x (PMC8481492; doi:10.1038/s41598-021-98405-x)

## Slide 1
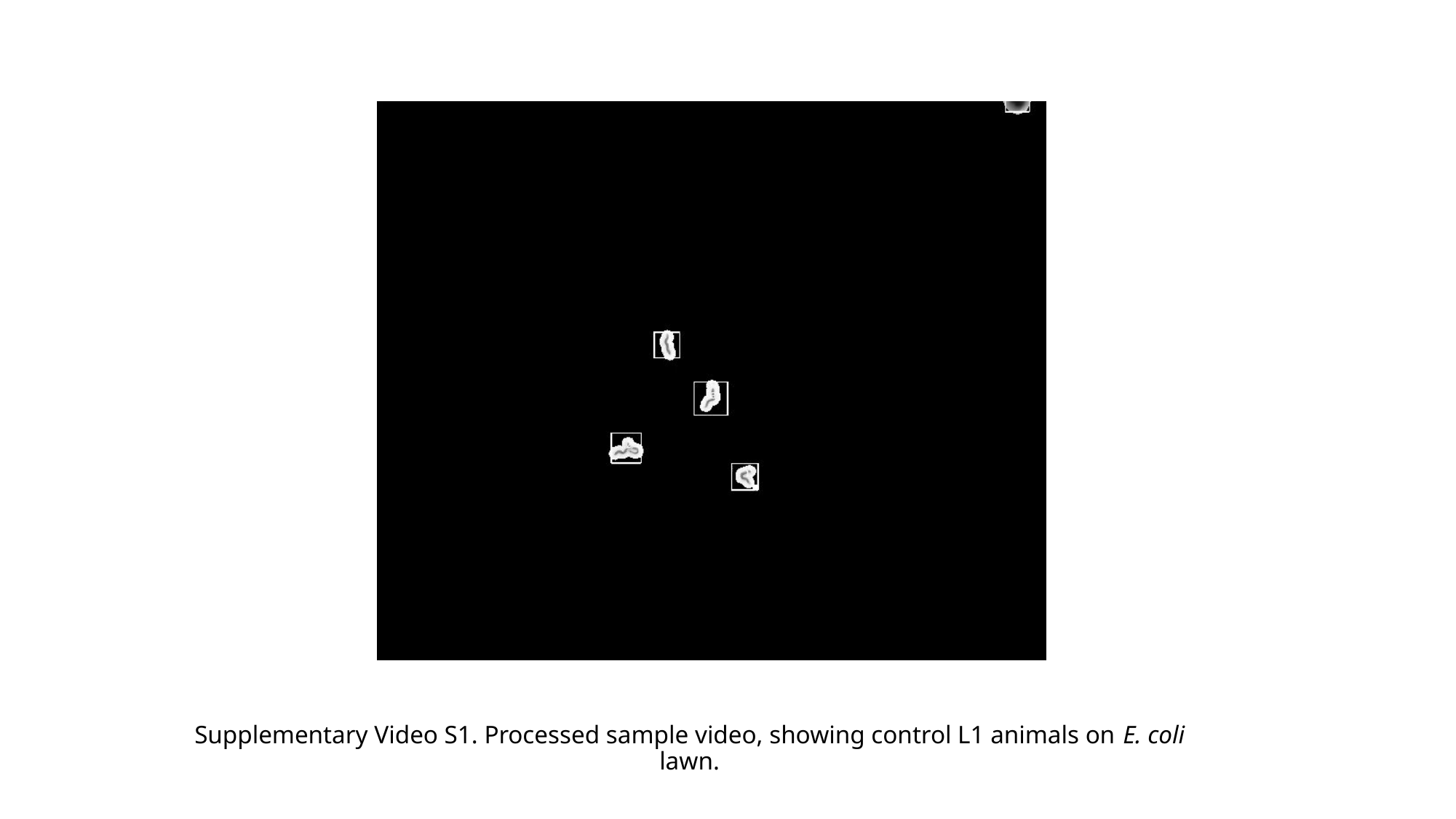

# Supplementary Video S1. Processed sample video, showing control L1 animals on E. coli lawn.

Supplement: Supplementary file 3 — Supplementary Video S1. [file 41598_2021_98405_MOESM3_ESM.pptx]
